# Maternal Diet Supplementation with n-6/n-3 Essential Fatty Acids in a 1.2 : 1.0 Ratio Attenuates Metabolic Dysfunction in MSG-Induced Obese Mice

**DOI:** 10.1155/2016/9242319

**Published:** 2016-12-05

**Authors:** Josiane Morais Martin, Rosiane Aparecida Miranda, Luiz Felipe Barella, Kesia Palma-Rigo, Vander Silva Alves, Gabriel Sergio Fabricio, Audrei Pavanello, Claudinéia Conationi da Silva Franco, Tatiane Aparecida Ribeiro, Jesuí Vergílio Visentainer, Elton Guntendeorfer Banafé, Clayton Antunes Martin, Paulo Cezar de Freitas Mathias, Júlio Cezar de Oliveira

**Affiliations:** ^1^Departamento de Biotecnologia, Genética e Biologia Celular, Universidade Estadual de Maringá, 87020-900 Maringá, PR, Brazil; ^2^Departamento de Química, Universidade Estadual de Maringá, 87020-900 Maringá, PR, Brazil; ^3^Universidade Tecnológica Federal do Paraná, 85902-490 Toledo, PR, Brazil; ^4^Instituto de Ciências da Saúde, Universidade Federal de Mato Grosso, 78557-267 Sinop, MT, Brazil

## Abstract

Essential polyunsaturated fatty acids (PUFAs) prevent cardiometabolic diseases. We aimed to study whether a diet supplemented with a mixture of n-6/n-3 PUFAs, during perinatal life, attenuates outcomes of long-term metabolic dysfunction in prediabetic and obese mice. Seventy-day-old virgin female mice were mated. From the conception day, dams were fed a diet supplemented with sunflower oil and flaxseed powder (containing an n-6/n-3 PUFAs ratio of 1.2 : 1.0) throughout pregnancy and lactation, while control dams received a commercial diet. Newborn mice were treated with monosodium L-glutamate (MSG, 4 mg g^−1^ body weight per day) for the first 5 days of age. A batch of weaned pups was sacrificed to quantify the brain and pancreas total lipids; another batch were fed a commercial diet until 90 days of age, where glucose homeostasis and glucose-induced insulin secretion (GIIS) as well as retroperitoneal fat and Lee index were assessed. MSG-treated mice developed obesity, glucose intolerance, insulin resistance, pancreatic islet dysfunction, and higher fat stores. Maternal flaxseed diet-supplementation decreased n-6/n-3 PUFAs ratio in the brain and pancreas and blocked glucose intolerance, insulin resistance, GIIS impairment, and obesity development. The n-6/n-3 essential PUFAs in a ratio of 1.2 : 1.0 supplemented in maternal diet during pregnancy and lactation prevent metabolic dysfunction in MSG-obesity model.

## 1. Introduction

Functional foods containing vitamins and/or antioxidants in addition to other substances are used to treat metabolic disorders and have been shown to mitigate metabolic syndrome progression [1, 2]. There is evidence supporting a role for foods containing essential polyunsaturated fatty acids (PUFAs), especially omega-3 (n-3) fatty acids, in preventing cardiometabolic diseases. The Western diet is known to contain high amounts of omega-6 (n-6) fatty acids and low levels of n-3 fatty acids [3]. High doses of n-6 fatty acids in the diet can cause metabolic dysfunction, induce the onset of obesity, and potentially induce cardiometabolic diseases [4, 5]. Diets with ratio of n-6/n-3 fatty acids close to 1 : 1 offered during pregnancy and lactation reportedly have beneficial effects on early brain development in the mice offspring [6].

Interestingly, disruption of the brain hypothalamus areas controlling body mass and energy metabolism is strongly involved in inducing several metabolic diseases in animal model [7]. Considering that metabolic dysfunctions have one of its possible origins in early life [8, 9], in the current work, we focus on the role of maternal diet enriched with essential PUFAs as a preventive tool against the metabolic syndrome. Among other obesity-models, the obesity induced by neonatal treatment with monosodium L-glutamate (MSG) is an interesting tool to study the effects of obesity and diabetic condition on different metabolic parameters. This model is characterized by hyperinsulinemia and higher insulin secretion associated with early hyperglycemia in MSG-treated mice [10–12]. MSG-induced obesity that is caused in neonatal rodents by partial destruction of the hypothalamus brain area which concentrates neuronal nuclei controlling body weight and energy metabolism has been long reported [13, 14]. All these dysfunctions in this model are essential to make the MSG-obese and prediabetic mice a good model to study metabolic diseases and its associated disturbances.

Using MSG-induced obesity in mice, in the present study we tested the hypothesis that perinatal diet supplemented with a mixture of n-6/n-3 fatty acids in a ratio of 1.2 : 1.0 supplied to lean dams, throughout pregnancy and lactation, can attenuate the onset of obesity in offspring treated with MSG during the first 5 days of life. In agreement with that, we assessed in the mice adult offspring the body composition and patterns regarding obesity and their related-complications such as impaired glucose homeostasis and pancreatic islets dysfunction.

## 2. Materials and Methods

### 2.1. Animals and Diets

The Ethical Committee for Animal Experiments at the State University of Maringa, which adheres to the Brazilian Federal Law, approved this protocol (protocol number 084/2009).

Virgin female mice aged 70 days with regular cycles were fed a commercial diet (Nuvital®, Curitiba, PR, Brazil) and maintained under controlled lighting and temperature (lights on from 07:00 to 19:00 h with temperature maintained at 23 ± 2°C) for acclimation. After that, virgin female mice were mated with proven male breeders and then the vaginal smear washed with saline solution (NaCl, 0.9%, w/v) was collected to evaluate the presence of spermatozoa to mark the first day of conception. After impregnation, females were kept in individual plastic cages and assigned to one of the two dietary treatments.

A batch of mice (*n* = 10) received a diet containing n-6 essential fatty acids from sunflower oil supplemented with flaxseed powder as source of n-3 fatty acids to reach an n-6/n-3 fatty acid ratio of 1.2 : 1.0 (Flax-diet, Table 1 and Table S1 in Supplementary Material available online at http://dx.doi.org/10.1155/2016/9242319) throughout pregnancy and lactation. While control mice (*n* = 10) were fed a commercial diet (Com-diet, Nuvital, Curitiba, PR, Brazil) throughout pregnancy and lactation. The composition of diets as well as the mixtures of salts and vitamins follows the recommendations of the AIN–93G [15].

Upon delivery, the litter size was adjusted to 6 pups per dam, preferentially male. Newborn mice from both dam groups (Flax-diet, *n* = 5 litters, and Com-diet, *n* = 5 litters) were subcutaneously injected with MSG (4 mg g^−1^ body weight once a day) in the cervical region within the first 5 days of life, while the other half of litters (*n* = 5) from each dam group was injected with an equimolar amount of saline solution. The male mice were kept, and all females were discarded at weaning (21st day). Thus, the number of 24 male mice was used as a standard number for each experimental offspring group, where a number between four and five mice, from each litter, were used. Indeed, as recommended, we used the number of litters (*n* = 5) for the statistical calculation in the present study [16]. MSG-treated offspring were divided into two groups: mice from mothers that received the flaxseed powder supplemented diet (MSG-Flax; *n* = 5) and mice from mothers fed the commercial diet (MSG-Com; *n* = 5).

The control offspring were divided into two additional groups: mice from mothers fed the flaxseed powder diet (Control-Flax; *n* = 5) and mice from mothers fed the commercial diet (Control-Com; *n* = 5). After weaning, the mice offspring were kept under controlled conditions with a temperature of 23 ± 2°C, a light cycle between 07:00 and 19:00 h, and water and commercial food* ad libitum*.

### 2.2. Quantification of Essential Fatty Acids Contained in Diet

The commercial diet contained n-6/n-3 fatty acids in a ratio of 10.7 : 1.0, while the sunflower oil diet supplemented with golden flaxseed flour used in this study contained n-6/n-3 fatty acids in a ratio of 1.2 : 1.0 (Table 2). Total lipids extraction and fatty acids methyl esters preparation were performed as described previously [17, 18]. The quantification of essential PUFAs in both Com-diet and Flax-diet was performed by gas chromatography (Thermo Scientific 3300, Waltham, MA, USA) fitted with a flame ionization detector and a fused-silica CP-7420 capillary column (100 m × 0.25 mm i.d. × 0.25 *μ*m of cyanopropyl polysiloxane). The operating parameters were 220°C temperature injection port, 240°C temperature detector, and 165°C column temperature for 12 min, which was programmed to increase at 40°C per min up to 180°C (hold time of 15 min, being raised to 240°C at 15°C per min with final holding time of 18.6 min). The carrier gas was hydrogen at 1.4 ml min^−1^; the nitrogen was used as the makeup gas at 30 ml min^−1^, with split injection at 1 : 80 ratio.

### 2.3. Evaluation of PUFAs Incorporation in the Brain and Pancreatic Tissue

To evaluate the incorporation of PUFAs in the brain and pancreatic tissues from weaned mice offspring, a batch of 21-day-old mice from all experimental groups (*n* = 5 per group) were sacrificed and brain and pancreas removed for lipids extraction. Lipid extraction was performed by the Bligh and Dyer method [17], as described above; thus, a volume of 500 *μ*l chloroform and 1000 *μ*l methanol were added per each 500 mg of tissue and the homogenate was subjected to posterior sonication (three times, 30 s pulses, using a Sonic Dismembrator Model 100; Fisher Scientific). After the lipid extraction and fatty acids methyl esters preparation, the quantification of essential fatty acids incorporated in the brain and pancreas tissues was performed in three replicates by gas chromatography, as described above. The quantification of fatty acids incorporation in the tissues (brain and pancreas) is depicted as *μ*g of fatty acids per mg of tissue. It was made against a C23:0 as internal standard purchased from Sigma-Aldrich® (Sigma-Aldrich, St. Louis, MO, USA), as described [19].

### 2.4. Intraperitoneal Glucose Tolerance Test (ipGTT)

The ipGTT was performed by intraperitoneally injecting glucose (2 g kg^−1^) into overnight-fasted mice. The number is 10 mice from 5 different litters (*n* = 5 for each group). The blood glucose levels were determined prior (0) to injection and 30, 60, 90, and 120 min after injection. All blood samples were obtained from the tail vein, and the plasma was used to measure the glucose concentration by the glucose oxidase method. The total area under the curve (AUC) for the ipGTT was calculated as previously reported [10].

### 2.5. Intraperitoneal Insulin Tolerance Test (ipITT)

In another batch of mice (*n* = 5 for each group), the ipITT was performed by intraperitoneally injecting insulin (1 UI kg^−1^ body weight) into the mice. The blood glucose levels were determined prior (0) to injection and 5, 15, 30, and 45 min after injection. All blood samples were obtained and the plasma glucose concentration was measured as described above. The constant rate for plasma glucose disappearance (*K*_itt_) was calculated using the formula 0.693(*t*_1/2_)^−1^. The plasma glucose half-life (*t*_1/2_) was calculated from the slope of the least squares analysis of the plasma glucose concentrations during the linear phase of decline after insulin injection in fed mice, as previously reported [20].

### 2.6. Pancreatic Islet Isolation and Insulin Secretion

Pancreatic islets were isolated using a collagenase technique as described previously [21], where three milliliters of Hanks buffered saline solution was injected into the mouse's common bile duct. Eight mice from at least 4 different litters were used for each experimental procedure per group (*n* = 4).

To adapt the isolated islets to a baseline glucose concentration (5.6 mmol l^−1^), the islets (four islets per well) were preincubated for 60 min with 1 ml of normal Krebs-Ringer solution [concentration in mmol l^−1^: NaCl, 115; NaHCO_3_, 24; KCl, 1.6; MgCl·6H_2_O, 1; CaCl_2_·2H_2_O, 1; and bovine serum albumin, BSA, 15] at pH 7.4 containing 5.6 mmol l^−1^ glucose. This solution was gassed with 95% O_2_ mixed with 5% CO_2_ to maintain a pH of 7.4. After the preincubation step, the islets were incubated with 2 different glucose concentrations, 5.6 and 16.7 mmol l^−1^, for an additional 60 min. The supernatants from these incubations were collected and stored for further insulin measurements by the radioimmunoassay method [22]. The limit of detection for insulin levels was 1.033 pmol l^−1^, as previously described [21].

### 2.7. Obesity Assessment

To evaluate the obesity parameters, at 90 days of age, after each one of the experimental procedures 18 mice from 5 different litter for all groups were used (*n* = 5). The retroperitoneal fat pad was removed and weighed to estimate obesity as previously reported [23]. The body length and weight were used to calculate the rodent body mass index, or Lee index [24].

### 2.8. Statistical Analyses

Data are presented as the mean ± SEM of the 5 different litters. All data were subjected to a D'Agostino-Pearson normality test to assess the Gaussian distribution. Afterward, all data were subjected to regular one-way ANOVA with a Bonferroni multiple comparisons posttest using GraphPad Prism software version 6.01 for Windows (GraphPad Software, Inc., San Diego, CA, USA). *P* values of <0.05 were considered statistically significant.

## 3. Results

### 3.1. Body Composition and Food Intake

**Figure 1:**
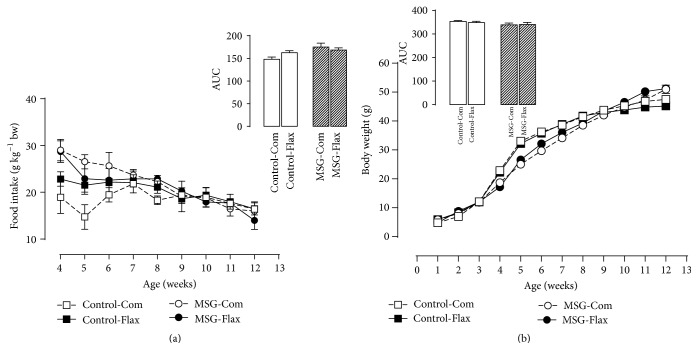
Effects of maternal flaxseed supplementation on offspring's food intake (a) and body weight progression (b). The data represent the mean ± SEM of 5 different litters (*n* = 5). The insets on each bar represent the area under the curve (AUC) from the food intake and body weight progression curves, respectively. The data were evaluated by one-way ANOVA.

Neither food intake nor body weight gain throughout period of life was affected by neonatal treatment with MSG and/or the maternal Flax-diet, as shown in Figures 1(a) and 1(b), respectively.

While treatment with the Flax-diet did not influence the Lee index in mice from the Control-Flax group, it was reduced by 8% in MSG-Flax mice, when compared to the MSG-Com group (*P* < 0.05; Table 2). Fat accretion in the retroperitoneal tissue was 3-fold higher in the MSG-Com group compared to the Control-Com group, *P* < 0.001, whereas it was decreased in both groups treated perinatally with the Flax-diet; fat accretion was reduced by 39% in the Control-Flax group and by 25% in the MSG-Flax group (*P* < 0.01; Table 2).

### 3.2. Glucose Homeostasis

**Figure 2:**
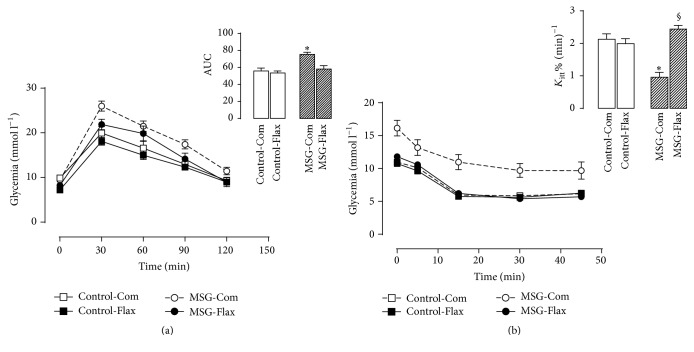
Effect of maternal flaxseed supplementation on offspring's glycemia during the intraperitoneal glucose (a) or insulin (b) tolerance tests. The symbols represent the mean ± SEM of 5 different litters (*n* = 5) from each experimental group. The inset on each bar represents the area under the curve (AUC) from glycemia during the glucose tolerance test and *K*_itt_ obtained from the insulin tolerance test, respectively. Symbol over the bar represents the significant differences among the groups based on one-way ANOVA, where *∗* depicts statistical difference with *P* < 0.001 among all groups and ^§^ *P* < 0.001 depicts statistical difference compared to MSG-Com group.

As expected, the MSG-Com treated mice exhibited hyperglycemia throughout the ipGTT, with an observed increase of 33% in the AUC compared to Control-Com mice (*P* < 0.001; Figure 2(a)). The maternal Flax-diet treatment, however, caused a 16% reduction in glucose intolerance compared to mice whose mothers did not receive n-6/n-3 fatty acids, allowing the MSG-Flax mice to normalize their glucose concentrations.

As showed in Figure 2(b), MSG-Com mice exhibited a low capacity for glucose uptake after insulin injection. The decrease in *K*_itt_ was 53% compared to Control-Com mice, *P* < 0.001. By contrast, the MSG-Flax treated mice displayed an increased glucose disappearance rate of 155%, *P* < 0.001. However, glucose uptake in the Control-Flax treated mice was not affected compared to the Control-Com treated mice.

### 3.3. Pancreatic Islets' Function on Secrete Insulin

**Figure 3:**
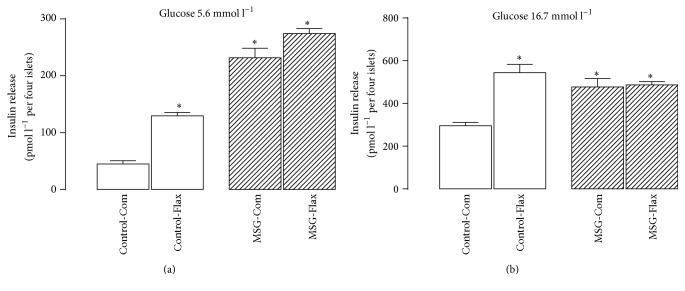
Effects of maternal flaxseed supplementation on insulin secretion stimulated by 5.6 mmol l^−1^ (a) or 16.7 mmol l^−1^ glucose (b). Bars represent the mean ± SEM of insulin secretion by pancreatic islets obtained from 8 mice of the 4 different litters (*n* = 4). The letters over the bars represent significant differences among the groups based on one-way ANOVA, where *∗* depicts statistical difference with *P* < 0.05 compared to Control-Com group.

Isolated pancreatic islets from the MSG-Com mice oversecreted insulin when stimulated with 5.6 mmol l^−1^ glucose; a 5-fold increase was observed compared to islets from Control-Com animals (*P* < 0.05; Figure 3(a)). Perinatal treatment with the Flax-diet increased glucose-induced insulin secretion (GIIS) up to 189% in the islets isolated from the Control-Flax mice group, while it was slightly increased by 18% in islets isolated from the MSG-Flax mice (*P* = 0.336; Figure 3(a)).

Glucose concentrations of 16.7 mmol l^−1^ also induced insulin oversecretion in islets isolated from the MSG-Com mice, a 2-fold increase in the GIIS compared to the Control-Com mice (*P* < 0.001). Perinatal treatment with the Flax-diet caused an 83%, *P* < 0.01, increase in GIIS that was stimulated by a high glucose concentration in the islets isolated from the Control-Flax group compared to the Control-Com group. By contrast, no change was observed in the MSG-Flax group (*P* > 0.999; Figure 3(b)).

### 3.4. Perinatal Brain and Pancreas Incorporation of Essential Polyunsaturated Fatty Acids

While the global brain incorporation of essential n-3 and n-6 PUFAs in the weaned MSG-treated mice was not impaired, it was increased by the maternal Flax-diet treatment in both control and MSG-treated mice (*P* < 0.01; Table S2, Supplementary Material).

Interestingly, the pancreas of weaned MSG-Com mice displayed less prominent incorporation of essential n-3 PUFAs associated with high incorporation of n-6 PUFAs in relation to Control-Com ones (*P* < 0.01; Table S3, Supplementary Material). Maternal Flax-diet treatment was able to induce an essential n-6 PUFAs reduction of 31% in eicosadienoic acid (EDA) and a reduction of 9% in arachidonic acid (AA) in Control-Flax, while it was reduced in 67% and 71%, respectively, in the MSG-Flax mice (*P* < 0.01; Table S3, Supplementary Material). On the other hand, the pancreatic n-3 PUFAs incorporation was increased by 320% to alpha-linolenic acid (LNA) and 229% to eicosapentaenoic acid (EPA) in Control-Flax mice, while in the MSG-Flax mice the pancreatic incorporation of the LNA and EPA was increased by 433% and 577%, respectively (*P* < 0.001; Table S3, Supplementary Material).

### 3.5. Ratio of n-6/n-3 Essential Polyunsaturated Fatty Acids in the Brain and Pancreas

**Figure 4:**
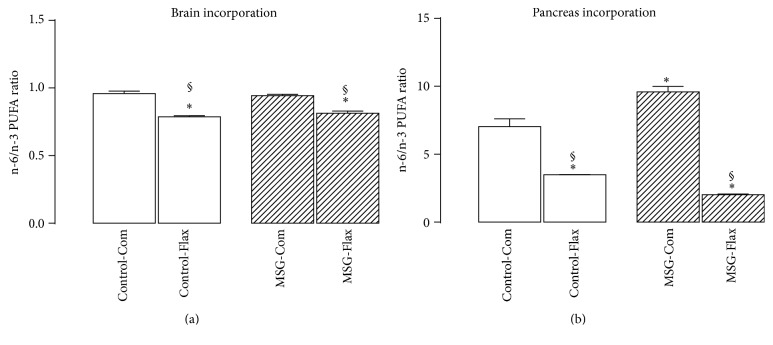
Polyunsaturated fatty acids (PUFAs) incorporation in the brain (a) and pancreatic (b) tissues from mice at 21 days of age. Bars represent the mean ± SEM of n-6/n-3 ratio for three replicates from samples obtained from 5 different litters (*n* = 5) of each group (*n* = 5). The letters over the bars represent significant differences among the groups based on one-way ANOVA, where *∗* depicts statistical difference with *P* < 0.001 compared to Control-Com group and ^§^ *P* < 0.001 depicts statistical difference compared to MSG-Com group.

Tissue incorporation of essential n-6/n-3 PUFAs ratio was examined in both brain and pancreas from weaned mice (Figure 4). While the neonatal treatment with MSG did not impair the n-6/n-3 essential PUFAs ratio in brain from weaned mice, when compared with control ones, perinatal treatment with Flax-diet induced a reduction in this ratio in the brain tissue from both control (–18%) and MSG-treated mice (–14%, *P* < 0.001; Figure 4(a)).

The n-6/n-3 essential PUFAs ratio incorporated in the pancreatic tissue from MSG-Com was 36% higher when compared with Control-Com group (*P* < 0.001; Figure 4(b)). Interestingly, the Flax-diet induced a reduction in both animal groups. However, the magnitude of improvement in the n-6/n-3 essential PUFAs ratio was strongly observed in the MSG-Flax mice (–79%), when compared with the Control-Flax mice (–50%, *P* < 0.001; Figure 4(b)).

## 4. Discussion

This study demonstrates that metabolic dysfunction is attenuated, or even blocked, by n-6/n-3 essential fatty acid (at a ratio of 1.2 : 1.0) supplementation in the maternal diet. To our knowledge, this is the first study showing that metabolic alterations, reflected by the Lee index, pancreatic islets dysfunction, and glucose intolerance, are restored in MSG-obese mice whose mothers were exposed to fatty acid supplementation during pregnancy and lactation. A well documented experimental model of cardiometabolic syndrome, the MSG-obesity model is characterized by hallmarks such as high fasting insulinemia, dyslipidemia, glucose intolerance, obesity, hypertension, and insulin resistance [25–28].

Once the regulation of growth hormone release in MSG-obese rodents is deficient due to a relative loss of growth hormone-releasing factor [29], the body weight in this model's rodents is lower than in their counterparts, even though the fat stores as well as Lee index are tightly huge compared to control ones, which is an unusual characteristic due to the lack of growth hormone in this experimental model. The growth hormone weakness is due to hypothalamic neuronal connection failure [30, 31]. Considering the early use of MSG injection in high concentrations, it acts as a powerful excitatory neurotransmitter by destroying the hypothalamic neuronal circuitry involved in the energy balance; it is the major mechanism behind the induction of obesity in this model. Once in early age the blood-brain barrier is not yet completed, the obesity-induction is promoted in neonatal rodents by partial destruction of the hypothalamus arcuate nucleus that contains neuronal circuitry controlling body weight and energy metabolism, as classically and long reported [13, 14, 32]. Indeed, beside the classical use of MSG as an obesity and metabolic syndrome model inductor in early life, it is important to keep in mind that there is a lot of misunderstanding about the MSG function on metabolism, especially regarding the dietary ingestion by adult human being (as recently revised [32]). In fact, glutamate food ingestion does not influence negatively the energy and/or metabolism homeostasis [33, 34]. Beyond umami-savory taste, through the basic taste function, the MSG from diet does not disturb brain or metabolic function, once it does not go to blood flow nor cross the blood-brain-barrier, thus not inducing obesity by this way [35].

Here we show a significant interaction pointing to amelioration of the metabolic dysfunction in MSG-obesity model associated with maternal diet supplementation with a mixture of n-6/n-3 fatty acids in a ratio of 1.2 : 1.0. According to our data, the Flax-diet treatment increased the insulin secretion in the islets isolated from control mice, while no change was observed in islets isolated from MSG-Flax mice. Interestingly, this finding may be associated with a mechanism of long-term protection against exhaustion by insulin oversecretion in the pancreatic islets of MSG-obese mice. In accordance with metabolic improvement, as observed in the present study, the incorporation of essential PUFAs, especially an increase in n-3, and a reduction in the n-6 essential PUFAs were higher in pancreatic tissue from weaned MSG-Flax mice that can reflect an important antioxidant role influencing the improvement in pancreatic function. Indeed, the beneficial effect of n-3 PUFAs in the cell membrane have been shown by displaying anti-inflammatory and antioxidant action as well as by protecting cells' metabolic function against the oxidative stress [36, 37].

Herein, we speculate that the high incorporation of n-3 and low n-6 essential fatty acids into pancreatic tissue from weaned mice, induced by Flax-diet, may be implicated in programming amelioration of pancreatic function in MSG-treated mice. Even if we did not have studied the beta-cells nor isolated pancreatic islets, our data can suggest lower ratio of n-6/n-3 essential PUFAs can exert some antioxidant action protecting the pancreatic islets metabolism and function against the oxidative stress. On this line, it is important to remark that pancreatic islet cells are important target of proinflammatory cytokines in obesity that induce beta-cell dysfunction [38].

Dietary supplementation with sunflower oil and golden flaxseed flour as sources of n-6/n-3 fatty acids in a 1.2 : 1.0 ratio in the maternal diet during pregnancy and lactation improved the metabolism of MSG-obese mice adult offspring, including a correction of glucose intolerance, low insulin sensitivity in peripheral tissues, high fat tissue accumulation, and insulin oversecretion. Maternal treatment with essential fatty acids was also able to improve the GIIS in the control mice. These data indicate that PUFAs are able to induce programming in the offspring that provides a certain resistance against metabolic deterioration. A previous study using a similar ratio of n-6/n-3 fatty acids in pregnant rats has shown that the offspring exhibited an improvement in early brain development [6], and amelioration in metabolic impairment has been reported to be induced by PUFAs dietary consumption [39, 40]. The data presented herein suggest that n-6/n-3 fatty acids in a ratio of 1.2 : 1.0 exert by somehow a sort of protection attenuating the deleterious central effects of MSG treatment. Unfortunately, we did not study the brain of offspring from mothers who were treated with the Flax-diet to assess, for instance, the arcuate nucleus structure or the expression of proteins involved in energy and metabolic control, which could suggest potential mechanisms underlying the resistance against obesity onset induced by maternal fatty acids treatment.

According to our results, high incorporation of n-3 taken together with low incorporation of n-6 essential PUFAs into brain tissue of weaned mice whose mothers were fed a Flax-diet during pregnancy and lactation is suggestive that this diet supplementation can prevent impairment in the hypothalamic central areas controlling body composition and energy balance, probably due to an action on reducing neuronal damage induced by MSG treatment early in life. Similar effects of n-3 essential PUFAs uptake in neurons from weaned mice of mothers fed a diet supplemented with flaxseed have been observed [6]. Indeed, the n-3 PUFAs in human diet have improved clinical symptoms regarding neurological impairment, which have been associated with beneficial implication in protecting against corticosterone-induced neuronal stress [41]. In this regard, synaptic plasticity, dopaminergic and serotonergic neurotransmission, and hypothalamic-pituitary-adrenal axis activity among many other neurological functions have been improved by n-3 PUFAs dietary supplementation [42–44]. As is known, hypothalamic-pituitary-adrenal axis hyperactivity is an important hallmark in obesity, like that in MSG-obesity model [27, 45], and in many other human's metabolic disorders [46–48].

In fact, as previously reported, the importance of n-3 PUFAs role against the inflammatory process in obesity and metabolic disturbances was showed in transgenic mice. High n-3/n-6 PUFAs ratio was strongly implicated in improving obesity-linked metabolic disturbances [39, 49]. Given that, it is remarkable that this amelioration has been associated with n-3 PUFAs action on preventing adipose inflammatory process [50], as well as on modulating changes in gut microbiota [39, 51], which is known to be strongly involved in obesity and metabolic syndrome development both in human and in animal model [52]. In this respect, on the fight against public health burden due to human's metabolic diseases around the world, the equilibrate n-6/n-3 ratio diet and/or seafood dietary ingestion is a healthy recommendation. It might be helpful and protect against the onset of metabolic syndrome in human population [39, 53, 54]. In the present study, beyond different possible mechanisms, it might also be a suggestive mechanism behind the metabolic amelioration observed in the MSG-obese mice.

In summary, our data show that dietary supplementation with n-6 and n-3 PUFAs in a ratio of 1.2 : 1.0 offered to mothers during pregnancy and lactation attenuates the metabolism malfunctions in MSG-obese mice adult offspring by improving pancreatic islets function, peripheral glucose tolerance, and insulin sensitivity as well reducing the fat mass stores. Considering that perinatal life is a fragile target to environmental aggression, which can induce metabolic syndrome later in life, early dietary supplementation with n-6 and n-3 PUFAs in a ratio of 1.2 : 1.0 can be powerfully used for protection against metabolic dysfunctions.

## Supplementary Material

In the supplementary material, are shown data about the amount and/or ration of essential polyunsaturated fatty acids (PUFAs) present in the maternal diet (Table S1), as well as the global incorporation of n-3 and n-6 PUFAs in the brain (Table S2) and pancreas (Table S3) from weaned mice.

## Figures and Tables

**Table 1:** Constituents of commercial diet (Com-diet) and diet supplemented with golden flaxseed flour and sunflower oil (Flax-diet). Values are given in g kg^−1^ of diet and the energy is given in kj kg^−1^. ^*∗*^Mixture of salts and vitamins used in the diet followed the recommendation of the AIN–93G [15].

Components	Com-diet		Flax-diet	
	(g)	(kj)	(g)	(kj)
Cornstarch	410.00	6.862	397.40	6.651
Casein	233.30	3.905	190.00	3.180
Dextrinated starch	89.20	1.493	101.70	1.702
Golden flaxseed	—	—	100.00	2.173
Sucrose	117.60	1.978	100.00	1.674
Sunflower oil	—	—	27.00	1.333
Fish oil	17.50	0.669	—	—
Soybean oil	48.50	1.836	—	—
Cellulose fibers	34.00	—	34.00	—
Minerals mix^*∗*^	30.00	—	30.00	—
Vitamins mix^*∗*^	15.00	—	15.00	—
L-Cystine	3.00	—	3.00	—
Choline bitartrate	1.90	—	1.90	—
Ascorbic acid	—	—	0.0014	—

Total	**1000.00**	**16.713**	**1000.00**	**16.713**

**Table 2:** Biometric parameters of mice at 90 days of age. Data are mean ± SEM of 18 mice from 3 different litters. The letters superscripts in each one of the values represent the significant differences among the groups by two-way ANOVA, where *∗* depicts statistical difference with *P* < 0.01 compared to Control-Com group and ^§^ *P* < 0.05 depicts statistical difference compared to MSG-Com group.

Biometric parameters	Control-Com	Control-Flax	MSG-Com	MSG-Flax
Body weight (g)	47.4 ± 0.65	45.2 ± 0.54	50.9 ± 1.44	51.2 ± 1.26
Retroperitoneal fat (g kg^−1^ bw)	1.17 ± 0.09	0.71 ± 0.02^*∗*§^	3.42 ± 0.17^*∗*^	2.25 ± 0.15^*∗*§^
Lee index	351.6 ± 2.8	343.6 ± 1.6^§^	388.3 ± 3.8^*∗*^	364.7 ± 3.2^*∗*§^

## Abbreviations

AA: Arachidonic acids
BSA: Bovine serum albumin
Control-Com: Group of control mice from mothers fed the commercial diet
Com-diet: Commercial diet
EDA: Eicosadienoic acids
EPA: Eicosapentaenoic acids
Flax-diet: Diet containing n-6 essential fatty acids from sunflower oil supplemented with flaxseed powder as source of n-3 fatty acids in a ratio of 1.2 : 1.0 for n-6/n-3 fatty acid
GIIS: Glucose-induced insulin secretion
HEPES: N-(2-Hydroxyethylpiperazine)-N′-2-ethanesulpho-nic acid
ipGTT: Intraperitoneal glucose tolerance test
ipITT: Intraperitoneal insulin tolerance test
LNA: Alpha-linolenic acids
*K*_itt_: Constant rate for plasma glucose disappearance
MSG-Flax: Group of MSG-treated mice from mothers fed Flax-diet
MSG: Monosodium L-glutamate
MSG-Com: Group of MSG-treated mice from mothers fed commercial diet
n-3: Omega-3
n-6: Omega-6
PUFAs: Polyunsaturated fat acids.
