# Supplementary material for: Maternal Diet Supplementation with n-6/n-3 Essential Fatty Acids in a 1.2 : 1.0 Ratio Attenuates Metabolic Dysfunction in MSG-Induced Obese Mice
Source: Int J Endocrinol. 2016 Dec 5;2016:9242319. doi: 10.1155/2016/9242319 (PMC5165167; doi:10.1155/2016/9242319)
Supplement: Supplementary file 1 — In the supplementary material, are shown data about the amount and/or ration of essential polyunsaturated fatty acids (PUFAs) present in the maternal diet (Table S1), as well as the global incorporation of n-3 and n-6 PUFAs in the brain (Table S2) and pancreas (Table S3) from weaned mice. [file 9242319.f1.pdf]

Supplementary material

Table S1 Ratio of essential fatty acids in commercial and golden flaxseed flour/sunflower oil supplemented diet.

**Fatty acids**	**Treatment (% of total lipids)**	
	**Com-diet**	**Flax-diet**
14:0	0.10 ± 0.00	0.25 ± 0.23
16:0	13.70 ± 0.06	6.15 ± 0.07
18:0	3.27 ± 0.02	4.15 ± 0.02
18:1 (n-9)	26.15 ± 0.23	26.19 ± 0.05
18:2 (n-6)	49.97 ± 0.24	33.85 ± 0.05
18:3 (n-3)	4.67 ± 0.15	28.11 ± 0.64
20:0	0.39 ± 0.03	0.19 ± 0.01
20:1 (n-9)	0.33 ± 0.01	0.18 ± 0.01
22:0	0.36 ± 0.02	0.40 ± 0.01
24:0	0.27 ± 0.01	0.17 ± 0.01
n-6/n-3 rate	10.70 ± 1.60	1.20 ± 0.08

Data are expressed as mean ± SEM of three replicates. Com-diet, commercial diet; Flax-diet, diet containing sunflower oil supplemented with flaxseed powder.

Table S2 Polyunsaturated fatty acids concentration in the brain tissue from mice at 21-day-old.

PUFAs (μg mg^{-1})	Treatment			
	Control-Com	Control-Flax	MSG-Com	MSG-Flax
LA, 18:2 (n-6)	0.18 ± 0.05	0.25 ± 0.02	0.21 ± 0.02	0.32 ± 0.05
LNA, 18:3 (n-3)	0.04 ± 0.01	0.10 ± 0.01 *	0.05 ± 0.01	0.08 ± 0.01 *
EDA, 20:2 (n-6)	0.05 ± 0.01	0.08 ± 0.00 *	0.06 ± 0.01	0.06 ± 0.01
DGLA, 20:3 (n-6)	0.10 ± 0.02	0.26 ± 0.04 *§	0.12 ± 0.01	0.20 ± 0.03 *§
AA, 20:4 (n-6)	1.62 ± 0.24	2.39 ± 0.15	2.08 ± 0.15	2.37 ± 0.24
EPA, 20:5 (n-3)	nq	0.02 ± 0.00	nq	0.03 ± 0.01
DPA, 22:5 (n-3)	0.02 ± 0.00	0.07 ± 0.00 *§	0.02 ± 0.00	0.09 ± 0.01 *§
DHA, 22:6 (n-3)	1.99 ± 0.32	3.38 ± 0.30 *	2.54 ± 0.18	3.41 ± 0.31 *

Data mean ± SEM for three replicate for each experimental group. Superscript symbols in the same line means statistical difference between the groups by one-way ANOVA, where * depicts statistical difference with $P < 0.05$ compared to Control-Com group and § depicts statistical difference with $P < 0.05$ compared to MSG-Com group. AA, arachidonic acid; LNA, alpha-linolenic acid; DGLA, dihomo-gamma-linolenic acid; DHA, docosahexaenoic acid; DPA, docosapentaenoic acid; EPA, eicosapentaenoic acid; EDA, eicosadienoic acid; LA, linoleic acid; . nq, no quantified (values < 0.01 μg mg^{-1} of tissue).

Table S3 Polyunsaturated fatty acids concentration in the pancreatic tissues from mice at 21-day-old.

PUFAs ($\mu g\ mg^{-1}$)	Treatment			
	Control-Com	Control-Flax	MSG-Com	MSG-Flax
LA, 18:2 (n-6)	5.45 ± 0.32	3.15 ± 0.87 *§	5.72 ± 0.56	3.78 ± 0.16 *§
LNA, 18:3 (n-3)	0.05 ± 0.01	0.21 ± 0.04 *	0.15 ± 0.04 *	0.80 ± 0.12 *§
EDA, 20:2 (n-6)	0.16 ± 0.02	0.11 ± 0.02 §	0.36 ± 0.04 *	0.12 ± 0.01 §
DGLA, 20:3 (n-6)	0.31 ± 0.03	0.45 ± 0.06 §	0.28 ± 0.03	0.21 ± 0.03
AA, 20:4 (n-6)	3.03 ± 0.41	2.75 ± 0.42 §	5.29 ± 0.23 *	1.53 ± 0.14 *§
EPA, 20:5 (n-3)	0.31 ± 0.03	1.02 ± 0.16 *§	0.13 ± 0.02 *	0.88 ± 0.07 *§
DPA, 22:5 (n-3)	0.24 ± 0.03	0.70 ± 0.08 *§	0.28 ± 0.02	0.50 ± 0.05 *§
DHA 22:6 (n-3)	0.44 ± 0.05	0.92 ± 0.09 *§	0.61 ± 0.05	0.63 ± 0.06

Data mean ± SEM for three replicate for each experimental group. Superscript symbols in the same line means statistical difference between the groups by one-way ANOVA, where * depicts statistical difference with $P < 0.05$ compared to Control-Com group and § depicts statistical difference with $P < 0.05$ compared to MSG-Com group. AA, arachidonic acid; LNA, alpha-linolenic acid; DGLA, dihomo-gamma-linolenic acid; DHA, docosahexaenoic acid; DPA, docosapentaenoic acid; EPA, eicosapentaenoic acid; EDA, eicosadienoic acid; LA, linoleic acid.
